# Evolutionary dynamics of GII.17 norovirus

**DOI:** 10.7717/peerj.4333

**Published:** 2018-02-01

**Authors:** Shaowei Sang, Xiaoyun Yang

**Affiliations:** 1 Clinical Epidemiology Unit, Qilu Hospital of Shandong University, Jinan, China; 2 Department of Gastroenterology, Qilu Hospital of Shandong University, Jinan, Shandong, China

**Keywords:** Norovirus, GII.17, Evolution

## Abstract

**Background:**

During the winter of 2014–2015, a rarely reported norovirus (NoV) genotype GII.17 was found to have increased its frequency in norovirus outbreaks in East Asia, surpassing the GII.4 NoV infections. GII.17 genotype has been detected for over three decades in the world. The aim of this study is to examine the evolutionary dynamics of GII.17 over the last four decades.

**Methods:**

NoV GII.17 sequences with complete or nearly complete VP1 were downloaded from GenBank and the phylogenetic analyses were then conducted.

**Results:**

The maximum likelihood analysis showed that GII.17 genotype could be divided into four different clades (Clades A–D). The strains detected after 2012, which could be the cause of the outbreaks, were separated into Clades C–D with their mean amino acid distance being 4.5%. Bayesian Markov chain Monte Carlo analyses indicated that the rate of nucleotide substitution per sites was 1.68 × 10^−3^ nucleotide substitutions/site/year and the time of the most recent common ancestor was 1840. The P2 subdomain of GII.17 was highly variable with 44% (56/128) amino acids variations including two insertions at positions 295–296 and one deletion at position 385 (Clades C and D) and one insertion at position 375 (Clade D). Variations existed in Epitopes A, B and D corresponding to GII.4 and human histo-blood group antigens binding site I in P2 subdomain.

**Conclusion:**

The novel GII.17 strains that caused outbreaks in 2013–2015 may have two new variants. The evolvement of HBGAs binding site and epitopes in P2 subdomain might contribute to the novel GII.17 strains predominance in some regions.

## Introduction

Human norovirus (NoV) is recognized as one of the most important causative agents of acute gastroenteritis in all age groups worldwide. Recent study estimated that the NoV prevalence in individuals with acute gastroenteritis was 18%, almost a fifth of the total acute-gastroenteritis cases (Ahmed et al., 2014). Whilst the impact of acute NoV infections is recognized worldwide, the long-term chronic NoV infections are more common in the immunocompromised population (Bok & Green, 2012).

NoV is a nonenveloped, positive-sense single stranded RNA virus with approximately 7.7 kb in length. The RNA genome is organized into three open reading frames (ORFs 1–3). ORF1 encodes a large nonstructural polyprotein. ORF2 and ORF3 encode major (VP1) and minor (VP2) capsid proteins, respectively. VP1 has a shell (S) and a protruding (P) domain which is further divided into P1 subdomain (residues 226–278 and 406–520) and the highly variable P2 subdomain (residues 279–405) (Prasad et al., 1999). At present, NoV is classified into seven genogroups based on the phylogenetic analysis of the complete VP1 amino acid sequences, and further divided into different genotypes (Vinje, 2015). Among them, the NoV GI and GII strains are commonly detected, which can be classified into nine and 22 genotypes, respectively (Kroneman et al., 2013).

Since the mid-1990s, epidemics of NoV gastroenteritis have been predominantly attributed to GII.4 genotype with periodic emergence of GII.4 variants every 2–3 years (Ramani, Atmar & Estes, 2014). In winter 2014–2015, a rarely reported NoV GII.17 genotype was found to have increased frequency in norovirus outbreaks in Southern China. This genotype contributed to 82% and 70% of the NoV outbreaks in Guangdong Province and Jiangsu Province, respectively (Fu et al., 2015; Lu et al., 2015). The novel GII.17 was also predominant in Hong Kong adjacent to Guangdong Province, and outcompeted the contemporary GII.4 Sydney 2012 variant (Chan et al., 2015). In addition to China, the novel GII.17 also emerged as a major cause of NoV outbreaks in Japan and South Korea in winter 2014–2015 (Dang Thanh et al., 2016; Matsushima et al., 2015). The novel GII.17 not only caused NoV outbreaks but also spread sporadically with cases reported in China (Shanghai, Taiwan) and USA during the winter of 2014–2015 (Chen et al., 2015; Lee et al., 2015; Parra & Green, 2015).

The viruses of GII.17 genotype have been detected for over three decades in the world (Rackoff et al., 2013). GII.17 NoV had been static before 2010. Before 2012, the GII.17 cases were sporadically reported in the world (De Graaf et al., 2015). From 2012 to 2013 GII.17 viruses accounted for 76% of all detected NoV strains along the rivers in rural and urban areas in Kenya (Kiulia et al., 2014). However, in the winter of 2014–2015, the novel GII.17 caused severe disease burden among NoV gastroenteritis in some regions of the world. Noroviruses undergo evolution through accumulations of point mutations and intragenotype or intergenotype recombination. Studying the genotypic variability of NoV is important to establish a phylogenetic map. Understanding the GII.17 genetic diversity and evolution pattern is very helpful to establish a more complete phylogenetic map, to learn about the mechanism of GII.17 epidemic, and to design vaccine against NoV. The aim of this study is to examine the evolutionary dynamics of GII.17 NoV over the last four decades.

## Materials and Methods

### Data

**Figure 1:**
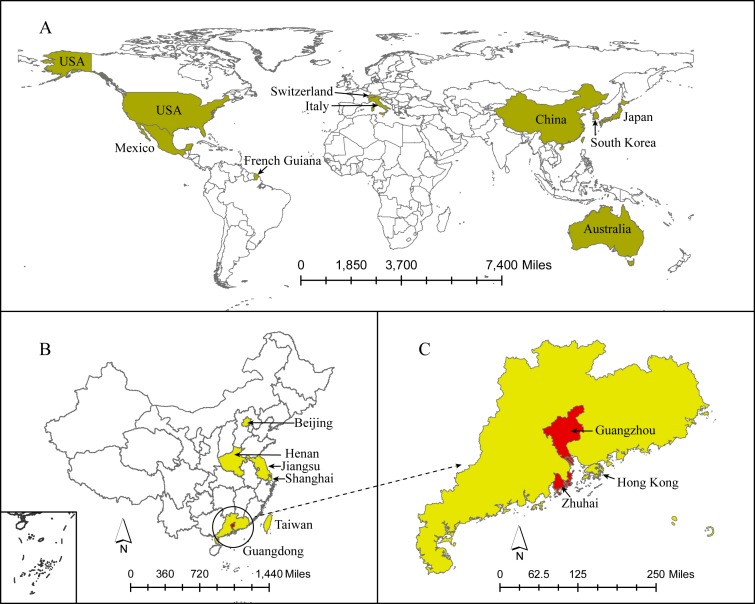
The locations with colours represented the regions where GII.17 strains were detected. (A–C) represented regions with GII.17 strains detected on the Country, Province and City level, respectively.

NoV GII.17 sequences were downloaded from GenBank. The search terms were “norovirus” AND “GII.17”. Only sequences with complete or nearly complete VP1 (>1,560 base pairs) were included for analyses. All the sequences were downloaded with the accession number, collection date and geographical region. One hundred and seventy-one GII.17 sequences were downloaded from GenBank (as of April 10th, 2016) (Table S1) (Fig. 1). The strains were named in the form of accession number/location/collection date (year). The collection date of some strains with 2014–2015 was the winter season of 2014–2015. Most strains were detected in the cold season.

### Phylogeny

Sequences alignments were performed using Multiple Alignment in Fast Fourier Transform (MAFFT) (Katoh, Asimenos & Toh, 2009). The parameters for the best-fit model of nucleotide substitutions were determined by Akaike information criterion (AIC) as implemented in jModelTest (Darriba et al., 2012). A phylogenetic tree was inferred by maximum-likelihood (ML) reconstruction as implemented with PHYML 3.0 Online (Guindon et al., 2010). The tree topology exploration was performed using subtree pruning and regrafting (SPR) algorithm. The reliability of branching pattern was tested through 100 bootstrap sampling. The tree was displayed with the FigTree program (http://tree.bio.ed.ac.uk/software/figtree/).

### Evolutionary analyses

The best-fit model of nucleotide substitutions was determined using AIC as implemented in jModelTest (Darriba et al., 2012). Rates of nucleotide substitution per sites and time to the most recent common ancestor (TMRCA) were estimated using Bayesian Markov chain Monte Carlo (MCMC) and implemented using the BEAST 1.8.2 software package (Drummond & Rambaut, 2007). Since no demographic model is available from organisms showing cyclic annual/seasonal behavior, the Bayesian Skyline model for population growth was chosen (Drummond et al., 2005). The calibration point for time is “year” when each strain was isolated, due to lack of exact collection date. Runs were performed under strict or relaxed (uncorrelated lognormal and exponential) clock model. Statistical uncertainty in parameter values was given by the 95% highest probability density (HPD) values. All chains were run sufficiently long to achieve convergence (the Effective Sample Size of continuous parameters greater than 200) after burn-in, as checked using TRACER 1.6 (http://tree.bio.ed.ac.uk/software/tracer/). As a result of the marginal likelihood calculation in the three clock, the dataset was analyzed using uncorrected exponential relaxed clock model (Table S2). The programs TreeAnnotator 1.8.2 and FigTree 1.4.2 were used to summarize the posterior tree distribution data produced by BEAST into a Maximum Clade Credibility (MCC) tree, where the branch length was calibrated to reflect temporal patterns and to visualize the annotated tree, respectively.

The amino acid variations within and between clades observed in the ML tree were examined by applying the Poisson correction with MEGA 6 (Tamura et al., 2013).

## Results

### Genetic diversity of GII.17

**Figure 2:**
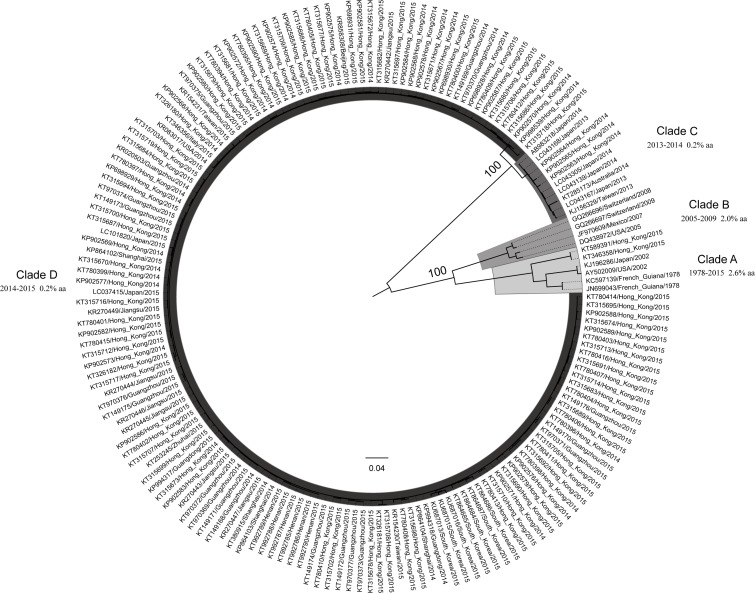
Maximum likelihood tree of full and nearly full VP1 region sequences from GII.17 NoV. The percent amino acid (aa) variation within each clades is shown.

Clades were defined as a minimum of three sequences of monophyletic origin. GII.17 genotype was divided into four different clades (Clades A–D) based on the maximum likelihood analysis. Strains in Clade A covered the longest time period ranged from 1978 to 2015. Clade B was composed of the strains detected from 2005 to 2009. Strains that were detected after 2012 were separated into Clade C and Clade D except two strains detected in Hong Kong in 2015. The intra-clade amino acid variation was 2.6%, 2.0%, 0.2% and 0.2%, respectively (Fig. 2). The mean amino acid distance between Clade C and Clade D was the smallest (4.5%) with the pairwise amino acid difference from 4.2% to 5.8% (Table 1).

**Table 1:** The mean amino acid distance between clades (minimum and maximum pairwise animo acid difference between clades).

	A	B	C	D
A	0			
B	8.6 (7.8, 9.4)	0		
C	10.8 (10.1, 11.5)	12.5 (12.1, 12.9)	0	
D	12.6 (11.8, 14.4)	14 (13.5, 14.4)	4.5 (4.2, 5.8)	0

### Evolutionary dynamics of GII.17 NoV

**Figure 3:**
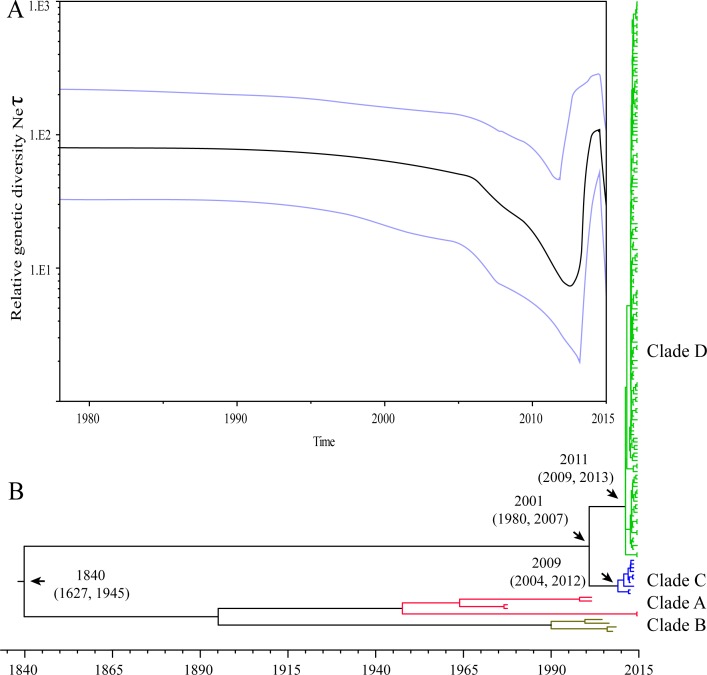
The results of Bayesian coalescent analyses. (A) depicted the changes in effective population size over time. The black line represents the median posterior value, and the blue lines the 95% HPD intervals; (B) showed the maximum credibility clade tree of GII.17 NoV VP1 sequences. For some nodes, the median estimated TMRCA and respective 95% Bayesian credible intervals were shown.

The demographic inference using Bayesian skyline plot (BSP) model is shown in Fig. 3A. The BSP from the capsid dataset shows that the GII.17 genetic diversity was nearly constant before 2005 and indicated peak for Neτ that coincided with the epidemic peak observed in winter 2014–2015. The uncorrelated exponential relaxed clock model indicated that GII.17 genotype evolved at a rate of 1.68 ×10^−3^ (95% HPD: 0.79 × 10^−3^ – 2.65 × 10^−3^) nucleotide substitutions/site/year. The phylogenetic analyses indicated that the TMRCA for the Clade C and D was 2009 (95% HPD interval: 2004–2012) and 2011 (95% HPD interval: 2009–2013), respectively. The divergent time for Clade C and D was estimated to be 2001 (95% HPD interval: 1980–2007). The TMRCA for GII.17 genotype was estimated to be 1840 (95% HPD interval: 1627–1945) (Fig. 3B).

As shown in Fig. S1, the P2 subdomain was highly variable with 44% (56/128) amino acids variations including two insertions at positions 295–296 and one deletion at position 385 (Clades C and D) and one insertion at position 375 (Clade D). Variations existed in Epitopes A, B and D corresponding to GII.4 and human histo-blood group antigens (HBGAs) binding site I in P2 subdomain.

## Discussion

Because of the worldwide predominance of the GII.4 NoV in epidemic gastroenteritis, previous studies had paid much attention to the mechanisms of GII.4 persistence in human populations (Lindesmith et al., 2008; Tan & Jiang, 2005). However, in the past two years the GII.17 has dominated the human NoV infections in some countries. This study downloaded the GII.17 sequences from GenBank and showed the dynamic evolution of GII.17 NoV. The study estimated that the GII.17 genotype has been circulated for over one century and a half in the world and the GII.17 strains diversity has been separated into four clades with human histo-blood group antigens (HBGAs) binding receptor and epitopes in the P2 subdomain evolvement over time.

The relative genetic diversity plot can help illustrate a demographical history by showing increases and decreases in genetic diversity within a defined time frame. BSP indicating the relative genetic diversity over time showed a marked change of GII.17 noroviruses. Literatures showed that GII.17 was prevalent in some countries or regions in the winter of 2014–2015 (Table S1) (Chan et al., 2015; Dang Thanh et al., 2016; Fu et al., 2015; Lu et al., 2015; Matsushima et al., 2015), but sporadically detected in the world before 2012 (De Graaf et al., 2015). The dynamics of GII.17 BSP were largely in agreement with the global GII.17 data, indicating the elevation in genetic diversity was consistent with the epidemiological data about the large-scale.

Because of the emergence of new pandemic GII.4 variants, GII.4 has been predominant since mid-1990s worldwide (Donaldson et al., 2008). In the absence of objective criteria, further subtyping into variants will be based on phylogenetic analysis and new variants are recognized only after evidence is provided that they have become the epidemic lineages in at least two geographically diverse locations (Kroneman et al., 2013). There were studies using large GII.4 dataset to explore the amino acid variations in VP1 region, and the results showed that strains in each variant had a maximum amino acid variation of 0.4 to 4.1% (Bok et al., 2009; Zheng et al., 2010). The GII.17 ML tree and MCC tree both had the same topology. The GII.17 dataset indicated that the strains in Clade C and D detected after 2012, which could cause outbreaks in different countries/regions, were genetically distinct and were strongly supported by the high bootstrap value. The minimum amino acid distance between Clade C and Clade D was 4.2%. Therefore, adapting to the same criterion for defining a new GII.4 variant (maximum amino acid variation of 4.1% within a variant), the evidences above suggested that GII.17 Clades C and D were two new GII.17 variants. The epidemiological information (Table S1) seems to suggestion that the Clade D variant displaced the Clade C variant in the 2014–2015 epidemic.

Data from 1975–2006 showed that GII.4 and GII.3 genotypes were the most common NoVs causing acute gastroenteritis outbreaks and sporadic cases, which might be attributed to their high evolutionary rate of VP1 capsid gene 4.3 × 10^−3^ and 4.16 × 10^−3^ nucleotide substitutions/site/year, respectively (Bok et al., 2009; Boon et al., 2011). Our study showed that the mean evolutionary rate of GII.17 was 1.68 × 10^−3^ nucleotide substitutions/site/year. This is comparable to the study conducted by Parra et al. (2017), which indicated that GII.17 noroviruses were a static genotype accumulating only a few amino acid mutations after decades of evolution. The estimates of GII.17 TMRCA reported by Matsushima et al. (1861; Matsushima et al., 2015) and by the present study (1840) also indirectly indicated that the evolutionary rate of GII.17 VP1 was possibly comparable. Whilst Chan et al. (2015) indicated that the mean evolutionary rate of GII.17 was 12 × 10^−3^ nucleotide substitutions/site/year, which was nearly three folds more than that of GII.4 VP1 capsid gene. However, the GII.4 genotype is much more diverse than GII.17. Markedly, the intra-clade amino acid variation of Clade A in GII.17 in our study was 2.6% among strains detected in 1978, 2002 and 2015 in different continents. Therefore, the VP1 gene in Clade A was highly conservative. Our results showed that the emerging GII.17 NoV (Clades C and D) were prevalent in China, South Korea and Japan, and sporadically detected in Europe, the United States and Australia (Fig. 2). These strains were highly similar and were detected in a short time period, suggesting that human movement might contribute to the emerging NoV to spread worldwide.

Human NoV recognize human histo-blood group antigens (HBGAs) as binding receptors (Donaldson et al., 2008). There were two HBGAs binding interfaces in P2 subdomain within GII group and the two HBGAs binding interfaces were highly conserved (Tan et al., 2009). The binding site I was variable in GII.17 strains with amino acid residuals replacements T349S and H353Q (Fig. S1), which made the first position of amino acid residual in Site I be the same with that of GII.4 strains. The GII.4 evolution was heavily influenced by antigenic variation of neutralizing epitopes (Lindesmith et al., 2012), which contributed to the 80% of human NoV infections. The emergence of GII.4 new pandemic strains is often associated with alternations in blockade epitopes mapped to the surface of the P2 subdomain (Donaldson et al., 2008). There were predicted four antibody epitopes in the P2 subdomain on the surface of GII.4. Corresponding to the positions of the four predicted epitopes in GII.4 NoV, variations existed in Epitope A, B and D of GII.17 strains. Epitope A including positions 294, 296–298, 368 and 372 is located on the top of the capsid proximal to the HBGAs binding pocket (Debbink et al., 2012). Of note, Epitope A is continuing to evolve in extent strains of GII.4 (Lindesmith et al., 2012), while it also evolved in GII.17 NoV in the study. Epitope D is comprised of three variable residues from position 393 to 395. The location of the Epitope on the surface of the capsid, directly proximal to the HBGA binding site (Shanker et al., 2011), likely plays a role in both receptor switching and in escape from herd immunity (Lindesmith et al., 2012). We speculate that the epitopes and HBGAs binding site evolvement could play great roles in GII.17 NoV prevalence in consideration of the mechanisms of GII.4 persistence.

The emerging GII.17 has caused widespread regional epidemics but not the whole world. It might be due to microbial make-up of the host or differences in the previous exposure of host populations to NoV (De Graaf, Van Beek & Koopmans, 2016). However, the mechanisms of GII.17 prevalence still need to be further studied.

The data was retrieved from Genbank and the GII.17 sequences were relatively few, especially sequences detected before 2010. Therefore, publication bias may exist with the GII.17 viruses of low virulence not reported. GenBank is an open access genetic sequence database and places no restrictions on the use or distribution of the data. The database updates periodically. Recently, we noticed a GII.17 strain deposited in GenBank on August 11th 2017 was detected in 1976 in Japan. We began to conduct the study and retrieved the sequences on 10th April 2016. Therefore, the sequences deposited to GenBank after the access date were not in consideration. The evidence power for the novel GII.17 strains to have caused outbreaks in 2013–2015 with possible two new variants was high because the amount of sequences detected in 2013–2015 was large. The calibration point using the exact month in the evolutionary analyses would be more reasonable. Unfortunately, we could not obtain the exact month of each strain collected. The relative genetic diversity result showed the dynamics of GII.17 were largely in agreement with the global GII.17 data based on the statistical models selected. Therefore, the “year” as the calibration point probably did not bias the statistical inference.

## Conclusion

In summary, the GII.17 strains can be divided into four clades based on genetic diversity of VP1. The strains caused outbreaks in 2013–2015 may be two new variants adapting to the same criterion for defining a new GII.4 variant. The amino acids of P2 subdomain were diverse with evolvement of HBGAs binding site and epitopes corresponding to GII.4, which could contribute to GII.17 prevalence in some regions. Given the sharp increase of GII.17 infections, it is necessary and important to monitor closely the new GII.17 variants before its spreading worldwide and to study the mechanism of GII.17 variants predominance.

##  Supplemental Information

10.7717/peerj.4333/supp-1 Figure S1 Amino acid variation of the P2 subdomain on the surface of VP1 capsid of representative strains over time Amino acid positions corresponding to GII.4 predicted antibody epitopes A–D were marked by symbols: “*”, Epitope A ; “”, Epitope B; “#”, Epitope C; “&”, Epitope D (Lindesmith et al., 2012). Site I and Site II are putative HBGAs binding sites of GII NoV (Tan et al., 2009). The amino acid sequences of the representative strains download from GenBank and aligned by Muscle in Mega 6.

10.7717/peerj.4333/supp-2 Table S1 Norovirus GII.17 download from GenBank

10.7717/peerj.4333/supp-3 Table S2 Model selection based on marginal likelihood estimates The best fitting model is ranked 1 (bold). BF, Bayes factors model selection; HM, Harmonic mean model selection; AICM, the Akaike information content model selection.

## Additional Information and Declarations

### Data Availability

The following information was supplied regarding data availability: All data was downloaded from GenBank.
