# Supplementary material for: Evolutionary dynamics of GII.17 norovirus
Source: PeerJ. 2018 Feb 1;6:e4333. doi: 10.7717/peerj.4333 (PMC5797681; doi:10.7717/peerj.4333)
Supplement: Figure S1 — Amino acid positions corresponding to GII.4 predicted antibody epitopes A–D were marked by symbols: “*”, Epitope A ; “”, Epitope B; “#”, Epitope C; “&”, Epitope D (Lindesmith et al., 2012). Site I and Site II are putative HBGAs binding sites of GII NoV (Tan et al., 2009). The amino acid sequences of the representative strains download from GenBank and aligned by Muscle in Mega 6. [file peerj-06-4333-s001.pdf]

	Clade		280								motif		290				*		*	*	*		300										310										320										330			@							#	341	
JN699043/French Guiana/1978	A	L	P	S	G	I	C	A	F	R	G	K	L	T	A	D	V	H	Q	S	H	D	D	R	W	H	M	Q	L	T	N	L	N	G	T	P	F	D	P	T	E	D	V	P	A	P	L	G	T	P	D	F	T	G	L	L	F	G	V	A	S	Q	R	N	V
AY502009/USA/2002	A	.	.	.	.	.	.	.	.	.	.	R	.	.	.	.	.	D	G	.	.	.	.	.	.	.	.	.	.	.	.	.	.	.	.	.	.	.	.	.	.	.	.	.	.	.	.	.	.	.	.	.	.	.	.	.	.	.	.	.	.	.	.	.	.
KJ196286/Japan/2002	A	.	.	.	.	.	.	.	.	.	.	R	.	.	.	.	.	D	G	.	.	.	.	.	.	.	.	.	.	.	.	.	.	.	.	.	.	.	.	.	.	.	.	.	.	.	.	.	.	.	.	.	.	.	.	.	.	.	.	.	.	.	.	.	.
KT346358/Hong Kong/2015	A	Q	.	.	.	.	.	.	.	.	.	.	I	.	.	.	.	R	P	N	.	.	.	.	.	.	.	.	.	.	.	.	.	.	.	.	.	.	.	.	.	.	.	.	.	.	.	.	.	.	.	.	.	.	.	.	.	.	.	.	.	.	.	.	.
DQ438972/USA/2005	B	.	.	T	.	.	.	.	.	.	.	.	I	S	.	.	.	Q	N	.	.	Q	.	.	.	.	.	.	.	.	.	.	.	.	.	.	.	.	.	.	D	.	.	.	.	.	.	.	.	.	.	.	.	.	.	.	.	.	.	.	.	.	.	.	.
LC043168/Japan/2013	C	.	.	.	.	.	.	.	.	.	.	R	V	.	.	E	T	-	-	D	.	R	.	K	.	.	.	.	.	Q	.	.	.	.	.	T	Y	.	.	.	D	.	.	.	.	.	.	.	.	.	.	.	K	.	V	V	.	.	.	.	.	.	.	.	.
AB983218/Japan/2014	C	.	.	.	.	.	.	.	.	.	.	R	V	.	.	E	T	-	-	D	.	R	.	K	.	.	.	.	.	Q	.	.	.	.	.	T	Y	.	.	.	D	.	.	.	.	.	.	.	.	.	.	.	K	.	V	V	.	.	.	.	.	.	.	.	.
KJ156329/Taiwan/2013	C	.	.	.	.	.	.	.	.	.	.	R	V	.	.	E	T	-	-	D	.	R	.	K	.	.	.	.	.	Q	.	.	.	.	.	T	Y	.	.	.	D	.	.	.	.	.	.	.	.	.	.	.	K	.	V	V	.	.	.	.	.	.	.	.	.
KP902563/Hong Kong/2014	C	.	.	.	.	.	.	.	.	.	.	R	V	.	.	E	T	-	-	D	.	R	.	K	.	.	.	.	.	Q	.	.	.	.	.	T	Y	.	.	.	D	.	.	.	.	.	.	.	.	.	.	.	K	.	V	V	.	.	.	.	.	.	.	.	.
KT285173/Australia/2014	C	.	.	.	.	.	.	.	.	.	.	R	V	.	.	E	T	-	-	D	.	R	.	K	.	.	.	.	.	Q	.	.	.	.	.	T	Y	.	.	.	D	.	.	.	.	.	.	.	.	.	.	.	K	.	V	V	.	.	M	.	.	.	.	.	.
KP698928/Hong Kong/2014	D	.	.	.	.	.	.	.	.	.	.	R	V	.	.	Q	I	-	-	N	Q	R	.	.	.	.	.	.	.	Q	.	.	.	.	.	T	Y	.	.	.	D	.	.	.	.	.	.	.	.	.	.	.	K	.	V	V	.	.	M	V	.	.	.	.	.
KP698930/Hong Kong/2015	D	.	.	.	.	.	.	.	.	.	.	R	V	.	.	Q	I	-	-	N	Q	R	.	.	.	.	.	.	.	Q	.	.	.	.	.	T	Y	.	.	.	D	.	.	.	.	.	.	.	.	.	.	.	K	.	V	V	.	.	M	V	.	.	.	.	.
KP864102/Shanghai/2015	D	.	.	T	.	.	.	.	.	.	.	R	V	.	.	Q	I	-	-	N	Q	R	.	.	.	.	.	.	.	Q	.	.	.	.	.	T	Y	.	.	.	D	.	.	.	.	.	.	.	.	.	.	.	K	.	V	V	.	.	M	V	.	.	.	.	.
KP864103/Shanghai/2014	D	.	.	.	.	.	.	.	.	.	.	R	V	.	.	Q	I	-	-	N	Q	R	.	.	.	.	.	.	.	Q	.	.	.	.	.	T	Y	.	.	.	D	.	.	.	.	.	.	.	.	.	.	.	K	.	V	V	.	.	M	V	.	.	.	.	.
KR020503/Guangzhou/2014	D	.	.	.	.	.	.	.	.	.	.	R	V	.	.	Q	I	-	-	N	Q	R	.	.	.	.	.	.	.	Q	.	.	.	.	.	T	Y	.	.	.	D	.	.	.	.	.	.	.	.	.	.	.	K	.	V	V	.	.	M	V	.	.	.	.	.
KT149170/Guangzhou/2015	D	.	.	.	.	.	.	.	.	.	.	R	V	.	.	Q	I	-	-	N	Q	R	.	.	.	.	.	.	.	Q	.	.	.	.	.	T	Y	.	.	.	D	.	.	.	.	.	.	.	.	.	.	.	K	.	V	V	.	.	M	V	.	.	.	.	.
KR083017/USA/2014	D	.	.	.	.	.	.	.	.	.	.	R	V	.	.	Q	I	-	-	N	Q	R	.	.	.	.	.	.	.	Q	.	.	.	.	.	T	Y	.	.	.	D	.	.	.	.	.	.	.	.	.	.	.	K	.	V	V	.	.	M	V	.	.	.	.	.
KR154230/Taiwan/2015	D	.	.	.	.	.	.	.	.	.	.	R	V	.	.	Q	I	-	-	N	Q	R	.	.	.	.	.	.	.	Q	.	.	.	.	.	T	Y	.	.	.	D	.	.	.	.	.	.	.	.	.	.	.	K	.	V	V	.	.	M	V	.	.	.	.	.
KR270442/Jiangsu/2015	D	.	.	.	.	.	.	.	.	.	.	R	V	.	.	Q	I	-	-	N	Q	R	.	.	.	.	.	.	.	Q	.	.	.	.	.	T	Y	.	.	.	D	.	.	.	.	.	.	.	.	.	.	.	K	.	V	V	.	.	M	V	.	.	.	.	.
KT253245/Zhuhai/2015	D	.	.	.	.	.	.	.	.	.	.	R	V	.	.	Q	I	-	-	N	Q	R	.	.	.	.	.	.	.	Q	.	.	.	.	.	T	Y	.	.	.	D	.	.	.	.	.	.	.	.	.	.	.	K	.	V	V	.	.	M	V	.	.	.	.	.
KT346356/Italy/2015	D	.	.	.	.	.	.	.	.	.	.	R	V	.	.	Q	I	-	-	N	Q	R	.	.	.	.	.	.	.	Q	.	.	.	.	.	T	Y	.	.	.	D	.	.	.	.	.	.	.	.	.	.	.	K	.	V	V	.	.	M	V	.	.	.	.	.
KT992785/Henan/2015	D	.	.	.	.	.	.	.	.	.	.	R	V	.	.	Q	I	-	-	N	Q	R	.	.	.	.	.	.	.	Q	.	.	.	.	.	T	Y	.	.	.	D	.	.	.	.	.	.	.	.	.	.	.	K	.	V	V	.	.	M	V	.	.	.	.	.
LC037415/Japan/2015	D	.	.	T	.	.	.	.	.	.	.	R	V	.	.	Q	I	-	-	N	Q	R	.	.	.	.	.	.	.	Q	.	.	.	.	.	T	Y	.	.	.	D	.	.	.	.	.	.	.	.	.	.	.	K	.	V	V	.	.	M	V	.	.	.	.	.

										Site I																													Site II																										
	Clade								350										360								*		370		*				#				380		@								390			&	&	&					400						
JN699043/French Guiana/1978	A	V	S	N	-	P	N	T	T	R	A	H	E	A	V	I	S	T	T	S	S	Q	F	V	P	K	L	G	S	I	N	F	G	-	S	T	S	D	D	F	Q	L	Q	Q	P	T	K	F	T	P	V	G	I	K	V	E	-	S	G	H	D	F	D	Q	W
AY502009/USA/2002	A	G	.	.	-	.	.	.	.	.	.	.	.	.	.	V	.	.	.	.	.	.	.	.	.	.	.	.	.	V	.	.	.	-	.	.	.	T	.	.	.	.	.	.	.	.	.	.	.	.	.	.	.	.	I	.	-	.	.	.	E	.	.	.	.
KJ196286/Japan/2002	A	G	.	.	-	.	.	.	.	.	.	.	.	.	.	.	.	.	.	.	.	.	.	.	.	.	.	.	.	V	.	.	.	-	.	.	.	T	.	.	.	.	.	.	.	.	.	.	.	.	.	.	.	.	I	.	-	.	.	.	E	.	.	.	.
KT346358/Hong Kong/2015	A	Q	P	.	-	.	.	.	.	.	.	.	.	.	.	.	.	.	.	.	.	.	.	.	.	.	.	.	.	.	.	.	.	-	.	.	.	E	.	.	I	V	G	.	.	.	.	.	.	.	.	.	.	.	I	.	-	D	.	.	A	.	.	.	.
DQ438972/USA/2005	B	G	G	T	G	N	.	.	.	.	.	.	.	V	.	.	A	.	.	.	T	.	.	.	.	.	.	.	.	.	.	.	.	-	.	E	.	E	.	.	.	V	G	P	.	.	.	.	.	.	.	.	.	.	I	.	-	T	.	.	S	.	R	.	.
LC043168/Japan/2013	C	G	N	D	A	.	G	S	.	.	.	.	.	.	.	.	.	.	Y	.	P	.	.	.	.	.	.	.	.	V	.	.	R	-	.	N	D	N	.	.	.	.	.	-	.	.	.	.	.	.	.	.	.	N	D	D	G	D	-	.	P	.	R	.	.
AB983218/Japan/2014	C	G	N	D	A	.	G	S	.	.	.	.	.	.	.	.	.	.	Y	.	P	.	.	.	.	.	.	.	.	V	.	.	R	-	.	N	D	N	.	.	.	.	.	-	.	.	.	.	.	.	.	.	.	N	D	D	G	D	-	.	P	.	R	.	.
KJ156329/Taiwan/2013	C	G	N	D	A	.	G	S	.	.	.	.	.	.	.	.	.	.	Y	.	P	.	.	.	.	.	.	.	.	V	.	.	R	-	.	N	D	N	.	.	.	.	.	-	.	.	.	.	.	.	.	.	.	N	D	D	G	D	-	.	P	.	R	.	.
KP902563/Hong Kong/2014	C	G	N	D	A	.	G	S	.	.	.	.	.	.	.	.	.	.	Y	.	P	.	.	.	.	.	.	.	.	V	.	.	R	-	.	N	D	N	.	.	.	.	.	-	.	.	.	.	.	.	.	.	.	N	D	D	G	D	-	.	P	.	R	.	.
KT285173/Australia/2014	C	G	N	D	A	.	G	S	.	.	.	.	.	.	.	.	.	.	Y	.	P	.	.	.	.	.	.	.	.	V	.	.	R	-	.	N	D	N	.	.	.	.	.	-	.	.	.	.	.	.	.	.	.	N	D	D	G	D	-	.	P	.	R	.	.
KP698928/Hong Kong/2014	D	G	N	D	A	.	G	S	.	.	.	Q	Q	.	W	V	.	.	Y	.	P	.	.	.	.	.	.	.	.	V	.	L	R	I	.	D	N	.	.	.	.	F	.	-	.	.	.	.	.	.	.	.	V	N	D	D	D	D	.	.	P	.	R	.	.
KP698930/Hong Kong/2015	D	G	N	D	A	.	G	S	.	.	.	Q	Q	.	W	V	.	.	Y	.	P	.	.	.	.	.	.	.	.	V	.	L	R	I	.	D	N	.	.	.	.	F	.	-	.	.	.	.	.	.	.	.	V	N	D	D	D	D	.	.	P	.	R	.	.
KP864102/Shanghai/2015	D	G	N	D	A	.	G	S	.	.	.	Q	Q	.	W	V	.	.	Y	.	P	.	.	.	.	.	.	.	.	V	.	L	R	I	.	D	N	.	.	.	.	F	.	-	.	.	.	.	.	.	.	.	V	N	D	D	D	D	.	.	P	.	R	.	.
KP864103/Shanghai/2014	D	G	N	D	A	.	G	S	.	.	.	Q	Q	.	W	V	.	.	Y	.	P	.	.	.	.	.	.	.	.	V	.	L	R	I	.	D	N	.	.	.	.	F	.	-	.	.	.	.	.	.	.	.	V	N	D	D	D	D	.	.	P	.	R	.	.
KR020503/Guangzhou/2014	D	G	N	D	A	.	G	S	.	.	.	Q	Q	.	W	V	.	.	Y	.	P	.	.	.	.	.	.	.	.	V	.	L	R	I	.	D	N	.	.	.	.	F	.	-	.	.	.	.	.	.	.	.	V	N	D	D	D	D	.	.	P	.	R	.	.
KT149170/Guangzhou/2015	D	G	N	D	A	.	G	S	.	.	.	Q	Q	.	W	V	.	.	Y	.	P	.	.	.	.	.	.	.	.	V	.	L	R	I	.	D	N	.	.	.	.	F	.	-	.	.	.	.	.	.	.	.	V	N	D	D	D	D	.	.	P	.	R	.	.
KR083017/USA/2014	D	G	N	D	A	.	G	S	.	.	.	Q	Q	.	W	V	.	.	Y	.	P	.	.	.	.	.	.	.	.	.	.	L	R	I	.	D	N	.	.	.	.	F	.	-	.	.	.	.	.	.	.	.	V	N	D	D	D	D	.	.	P	.	R	.	.
KR154230/Taiwan/2015	D	G	N	D	A	.	G	S	.	.	.	Q	Q	.	W	V	.	.	Y	.	P	.	.	.	.	.	.	.	.	V	.	L	R	I	.	D	N	.	.	.	.	F	.	-	.	.	.	.	.	.	.	.	V	N	D	D	D	D	.	.	P	.	R	.	.
KR270442/Jiangsu/2015	D	G	N	D	A	.	G	S	.	.	.	Q	Q	.	W	V	.	.	Y	.	P	.	.	.	.	.	.	.	.	V	.	L	R	I	.	D	N	.	.	.	.	F	.	-	.	.	.	.	.	.	.	.	V	N	D	D	D	D	.	.	P	.	R	.	.
KT253245/Zhuhai/2015	D	G	N	D	A	.	G	S	.	.	.	Q	Q	.	W	V	.	.	Y	.	P	.	.	.	.	.	.	.	.	V	.	L	R	I	.	D	N	.	.	.	.	F	.	-	.	.	.	.	.	.	.	.	V	N	D	D	D	D	.	.	P	.	R	.	.
KT346356/Italy/2015	D	G	N	D	A	.	G	S	.	.	.	Q	Q	.	W	V	.	.	Y	.	P	.	.	.	.	.	.	.	.	V	.	L	R	I	.	D	N	.	.	.	.	F	.	-	.	.	.	.	.	.	.	.	V	N	D	D	D	D	.	.	P	.	R	.	.
KT992785/Henan/2015	D	G	N	D	A	.	G	S	.	.	.	Q	Q	.	W	V	.	.	Y	.	P	.	.	.	.	.	.	.	.	V	.	L	R	I	.	D	N	.	.	.	.	F	.	-	.	.	.	.	.	.	.	.	V	N	D	D	D	D	.	.	P	.	R	.	.
LC037415/Japan/2015	D	G	N	D	A	.	G	S	.	.	.	Q	Q	.	W	V	.	.	Y	.	P	.	.	.	.	.	.	.	.	V	.	L	R	I	.	D	N	.	.	.	.	F	.	-	.	.	.	.	.	.	.	.	V	N	D	D	D	D	.	.	P	.	R	.	.
